# Genetic polymorphism of IDOL gene was associated with the susceptibility of coronary artery disease in Han population in Xinjiang, China

**DOI:** 10.1186/s41065-021-00178-w

**Published:** 2021-04-12

**Authors:** Dilare Adi, Jialin Abuzhalihan, Jing Tao, Yun Wu, Ying-Hong Wang, Fen Liu, Yi-Ning Yang, Xiang Ma, Xiao-Mei Li, Xiang Xie, Zhen-Yan Fu, Yi-Tong Ma

**Affiliations:** 1 Department of Cardiology, First Affiliated Hospital of Xinjiang Medical University, Urumqi, 830054 P.R. China; 2 Xinjiang Key Laboratory of Cardiovascular Disease Research, Urumqi, 830054 P.R. China; 3 People’s Hospital of Xinjiang Uygur Autonomous Region, Urumqi, 830001 P.R. China; 4 Department of General Practice, First Affiliated Hospital of Xinjiang Medical University, Urumqi, 830054 P.R. China; 5 Health Checkup Department of The First Affiliated Hospital of Xinjiang Medical University, Urumqi, 830054 P.R. China; 6 State Key Laboratory of Pathogenesis, Prevention and Treatment of High Incidence Diseases in Central Asia, Clinical Medical Research Institute, the First Affiliated Hospital of Xinjiang Medical University, Urumqi, 830054 P.R. China

**Keywords:** IDOL, Polymorphism, Coronary artery disease

## Abstract

**Background:**

Coronary artery disease (CAD) is the leading cause of death worldwide. In this study, we aimed to explore whether some genetic variants of the human IDOL gene were associated with CAD among Chinese population in Xinjiang.

**Methods:**

We designed two independent case–control studies. The first one included in the Han population (448 CAD patients and 343 controls), and the second one is the Uygur population (304 CAD patients and 318 controls). We genotyped three SNPs (rs2072783, rs2205796, and rs909562) of the IDOL gene.

**Results:**

Our results revealed that, in the Han female subjects, for rs2205796, the distribution of alleles, dominant model (TT vs. GG + GT) and the additive model (GG + TT vs. GT) showed significant differences between CAD patients and the control subjects (*P* = 0.048, *P* = 0.014, and *P* = 0.032, respectively).

**Conclusions:**

The rs2205796 polymorphism of the IDOL gene is associated with CAD in the Chinese Han female population in Xinjiang, China.

## Background

Coronary artery disease (CAD), also known as coronary heart disease (CHD), is one of the leading causes of death worldwide. Atherosclerosis and its complications, including coronary atherosclerotic heart disease and cerebral atherosclerotic vascular disease, account for nearly 80% of CAD deaths [1–3]. Certain risk factors, including increased plasma low-density lipoprotein (LDL), triglyceride (TG) levels, smoking, obesity, hypertension, diabetes, aging etc. increase the incidence of atherosclerosis [4, 5]. In recent years, with the development of genome technology, researchers have found that several genetic susceptibility genes and the risks of CAD were associated in several ways.

IDOL, also known as myosin regulatory light chain interacting protein (MYLIP), is a liver X receptor-target gene: increased cellular cholesterol levels activate liver X receptor and increase IDOL expression [6]. In addition, by mediating the ubiquitination of the intracellular tail of the receptor and its lysosomal degradation, IDOL could control the LDLR abundance [7]. Cells lacking IDOL showed significantly increased LDLR protein levels, sterol depleted growth conditions and increased LDL uptake under basal. Further, IDOL-null cells fail to respond to synthesized LXR ligands and down regulate LDLR levels [8, 9]. Therefore, IDOL is a pivotal gene in the process of cholesterol metabolism.

Experiments related hepatic overexpression of the IDOL gene in mouse showed hypercholesterolemia and development of atherosclerosis [10, 11]. The human IDOL gene is identified as an important gene in cholesterol metabolism by Genome-wide association studies (GWAS) [12, 13]. Studies concerning the relationships between human IDOL gene and CAD remain controversial results. A study in a Mexican population showed that the rs9370867 single nucleotide polymorphism (SNP) on human IDOL gene was correlated with high total cholesterol (TC) levels [14]. Another study in a Dutch population showed that there was no difference in IDOL gene rs9370867 polymorphism between two populations with low levels of low-density lipoprotein-cholesterol (LDL-C) and high levels of LDL-C [15]. Nevertheless, an investigation in a Brazilian population showed that there were no associations between the IDOL gene rs9370867 SNP and lipid profiles [16]. Furthermore, the relevance of CAD and human IDOL gene remains unclear. Xinjiang is located in the northwest of China, where more than forty ethnic groups live in here. There are few intermarriages among these ethnic groups, and the characteristics of each ethnic group are basically maintained. Uygur and Han populations account for 48.53 and 40.1% of them, respectively. Compared with other minority ethnic groups, Uygur people has a large population, which is comparable with the Han population. Therefore, the two ethnic groups were included in our study. Our main objective is to analyze the correlation between some polymorphisms of the human IDOL gene and CAD in Uygur and Han population in Xinjiang, China.

## Methods

### Ethical approval of the study protocol

The study was approved by the Ethics Committee of the First Affiliated Hospital of Xinjiang Medical University. All participants signed an informed consent. The investigation was conducted in accordance with the principles of the Helsinki declaration.

### Population sample

The study was carried out in two independent case–control study designs. All participants were selected from the First Affiliated Hospital of Xinjiang Medical University from August 2013 to October 2019. We selected 448 Chinese Han patients (333 men and 115 women) diagnosed with CAD. Meanwhile, 304 Chinese Uygur CAD patients (247 men and 57 women) were recruited between the same period of time. According to the coronary angiography, CAD was defined that the presence of at least one significant coronary artery stenosis has over 50% luminal diameter. For two CAD groups, participants, not suffering from any diseases and matched for ethnicity, sex, and ages, were selected into the control groups. The subjects [Han, *n* = 343 (165 men and 178 women) and Uygur, *n* = 318 (139 men and 179 women)] in control groups were selected from healthy volunteers who had normal coronary angiography. Exclusion criteria included those suffering from impaired malignancy, connective tissue disease, concomitant valvar heart disease, chronic inflammatory disease or valvular disease, renal function, pancreatic disease, thyroid disease, fatty liver, cirrhosis, hepatitis. Hypertension was defined as a systolic blood pressure ≥ 140 mmHg and/or a diastolic blood pressure ≥ 90 mmHg of both arms on three consecutive measurements in different days [17]. Diabetes mellitus was diagnosed when two consecutive measurements on plasma glucose level ≥ 11.1 mmol/L and/or fasting plasma glucose levels ≥ 7.0 mmol/L two hours after meal [18]. The information, including hypertension, diabetes, age, gender, total cholesterol (TC), triglyceride (TG), low-density lipoprotein cholesterol (LDL-C) and high-density lipoprotein cholesterol (HDL-C), was collected.

### Genotyping

Using phase I&II database on the International HapMap Project website and Haploview 4.2 software, three tag SNPs of the IDOL gene: SNP1 (rs2072783), SNP2 (rs2205796), and SNP3 (rs909562) were selected with linkage disequilibrium patterns (*r*^2^ ≥ 0.8) and minor allele frequency (MAF ≥ 0.05). Blood samples were collected from all subjects. With the use of a DNA extraction kit developed by Beijing Biotech Co. Ltd, Genomic DNA was extracted from peripheral vein blood leukocytes. SNP genotyping was performed based on the iMLDR (improved multiplex ligation detection reaction). Genotyping was carried out by blinded method without knowing any clinical data of patients, and some genotyped samples (10%) were repeated to monitor genotyping quality.

### Statistical analysis

Statistical analyses were carried out by SPSS version 22.0 (SPSS, Chicago, IL). All data from the SPSS were used for the variance tests of equal and normality (Kolmogorov–Smirnov test). Continuous variables are expressed as mean ± SD in case of normal distribution and the median (minimum to maximum) in case of non-normal distribution. Quantitative variables were compared with independent-Sample T-test, and the chi-squared test was used to analyze the differences in qualitative data (drinking, smoking and IDOL genotypes) taken from the case and control groups. Finally, logistic regression analysis was used to assess the major risk factors of CAD, and a *P* value < 0.05 was considered statistically significant.

## Results

### Characteristics of study participants

Table 1 showed the clinical and metabolic characteristics of the two study populations. For both two groups, the CAD patients’ average age was higher than control subjects, and the prevalence of smoking, diabetes, hypertension, and drinking in patients with CAD was higher than control groups. CAD patients have significantly higher levels of LDL-C, TC, FPG and lower levels of HDL-C than control subjects in both Han and Uygur groups. Uygur patients also have significantly higher levels of TG and high sensitive C reactive protein (hs-CRP) than the control group.

**Table 1:** Baseline characteristics of study population

**Groups**	Age, years	Male, n (%)	Smoking, n (%)	Drinking, n (%)	Hypertension, n (%)	Diabetes, n (%)	TG, mmol/L	TC, mmol/L	HDL-C, mmol/L	LDL-C, mmol/L	FPG, mmol/L	hs-CRP, mg/L	CK, IU/L	ApoA, g/L	ApoB, g/L
Han															
CAD group	61.26 ± 10.99	333 (74.3%)	224 (50.0%)	243 (54.2%)	257 (57.4%)	116 (25.9%)	2.03 ± 1.45	4.86 ± 0.86	1.00 ± 0.33	3.05 ± 1.34	6.33 ± 2.63	2.02 ± 0.72	91.52 ± 8.08	1.24 ± 0.31	0.83 ± 0.40
Control group	56.11 ± 12.86	165 (48.1%)	116 (33.8%)	95 (27.7%)	140 (40.8%)	41 (12.0%)	1.87 ± 1.54	4.15 ± 1.11	1.26 ± 0.58	2.53 ± 0.96	5.70 ± 2.00	1.99 ± 0.67	90.87 ± 10.64	1.22 ± 0.49	0.81 ± 0.24
χ^2^ or t	6.067	57.292	20.753	55.934	21.284	23.728	1.487	9.901	8.068	6.346	3.736	0.521	0.961	0.702	0.881
* P* value	< 0.001	< 0.001	< 0.001	< 0.001	< 0.001	< 0.001	0.137	< 0.001	< 0.001	< 0.001	< 0.001	0.602	0.337	0.483	0.379
Uygur															
CAD group	56.07 ± 9.57	247 (81.3%)	129 (42.4%)	137 (45.1%)	177 (58.2%)	70 (23.0%)	1.97 ± 1.15	4.49 ± 1.11	0.91 ± 0.60	2.92 ± 1.43	6.16 ± 2.53	2.21 ± 0.71	89.97 ± 6.82	1.14 ± 0.25	0.83 ± 0.25
Control group	52.67 ± 8.61	139 (43.7%)	66 (20.8%)	28 (8.8%)	148 (46.5%)	28 (8.8%)	1.63 ± 1.07	4.21 ± 1.38	1.21 ± 0.47	2.65 ± 1.32	5.12 ± 2.74	2.10 ± 0.63	88.72 ± 9.25	1.12 ± 0.29	0.80 ± 0.26
χ^2^ or t	4.719	93.017	33.941	104.849	8.502	23.682	3.706	2.758	6.968	2.377	6.021	2.169	1.893	1.35	0.29
* P* value	< 0.001	< 0.001	< 0.001	< 0.001	0.004	< 0.001	< 0.001	0.006	< 0.001	0.018	< 0.001	0.03	0.059	0.178	0.197

Data are presented as Number of patients (%) or mean standard ± deviation

*Note*: For both Han and Uygur groups, the mean age of patients with CAD was higher than control group’. The prevalence of smoking, diabetes, hypertension, and drinking was higher in case group. LDL-C, TC, FPG levels were higher in case group. HDL-C levels were lower in case group. The TG and hs-CRP levels were higher in Uygur CAD patients

*TC* total cholesterol, *TG* triglyceride, *HDL-C* high-density lipoprotein cholesterol, *LDL-C* low-density lipoprotein cholesterol, *FPG* fasting plasma glucose, *hs-CRP* high sensitive C reactive protein, *CK* creatine kinase, *ApoA* Apolipoprotein A, *ApoB* Apolipoprotein B

### Distributions of genotype and alleles in subjects

Tables 2, 3, 4 and 5 separately showed the genotypes and alleles distribution for three SNPs (rs2072783, rs2205796, rs909562) of the IDOL gene in Han and Uygur populations. The genotype distributions of these SNPs met the Hardy–Weinberg equilibrium balance (all *P* > 0.05). Our results showed that, in the Han male participants, there were no significant differences in the distributions of genotypes and alleles, dominant model, recessive model, and additive model for three SNPs between case and control groups (all *P* > 0.05). However, in the Han female subjects, for rs2205796, there were significant differences in the genotypes and alleles distribution, dominant model (TT vs. GG + GT) and additive model (GG + TT vs. GT) between the case and control groups (*P* = 0.048, *P* = 0.019, *P* = 0.014, and *P* = 0.032, respectively; Table 3). Nevertheless, the three SNPs and alleles distribution did not show differences in Uygur male and female participants.

**Table 2:** Distribution of SNPs of IDOL gene in Han male subjects

**Genotype**	**Model**		**Case (n, %)**	**Control (n, %)**	***P value***
**rs2072783** (A > G)	Codominant	AA	120 (36.0)	55 (33.3)	0.838
		GA	157 (47.1)	81 (49.1)	
		GG	56 (16.8)	29 (17.6)	
	Dominant	AA	120 (36.0)	55 (33.3)	0.552
		GA + GG	213 (64.0)	110 (66.7)	
	Recessive	AA + GA	277 (83.2)	136 (82.4)	0.832
		GG	56 (16.8)	29 (17.6)	
	Additive	GG + AA	176 (52.9)	84 (50.9)	0.683
		GA	157 (47.1)	81 (49.1)	
		A	397 (62.4)	191 (57.9)	0.601
		G	269 (37.6)	139 (42.1)	
**rs2205796** (T > G)	Codominant	TT	198 (59.5)	104 (63.0)	0.283
		GT	124 (37.2)	52 (31.5)	
		GG	11 (3.3)	9 (5.5)	
	Dominant	TT	19,859.5)	104 (63.0)	0.443
		GT + GG	135 (40.5)	61 (37.0)	
	Recessive	TT + GT	322 (96.7)	156 (94.5)	0.250
		GG	11 (3.3)	9 (5.5)	
	Additive	GG + TT	209 (62.8)	113 (68.5)	0.209
		GT	124 (37.2)	52 (31.5)	
		T	520 (78.1)	260 (78.8)	0.798
		G	146 (21.9)	70 (21.2)	
**rs909562** (A > G)	Codominant	AA	134 (40.2)	55 (33.3)	0.316
		GA	151 (45.3)	82 (49.7)	
		GG	48 (14.4)	28 (17.0)	
	Dominant	AA	134 (40.2)	55 (33.3)	0.135
		GA + GG	199 (59.8)	110 (66.7)	
	Recessive	GA + AA	285 (85.6)	137 (83.0)	0.455
		GG	48 (14.4)	28 (17.0)	
	Additive	GG + AA	182 (54.7)	83 (50.3)	0.360
		GA	151 (45.3)	82 (49.7)	
		A	419 (62.9)	192 (58.2)	0.149
		G	247 (37.1)	138 (41.8)	

*Note*: In the Han male participants, there were no significant differences in the distributions of genotypes and alleles, dominant model, recessive model, and additive model for three SNPs between case and control groups (all *P* > 0.05)

**Table 3:** Distribution of SNPs of IDOL gene in Han female subjects

**Genotype**	**Model**		**Case (n, %)**	**Control (n, %)**	***P value***
**rs2072783** (A > G)	Codominant	AA	42 (36.5)	67 (37.6)	0.283
		GA	60 (52.2)	80 (44.9)	
		GG	13 (11.3)	31 (17.4)	
	Dominant	AA	42 (36.5)	67 (37.6)	0.847
		GA + GG	73 (63.5)	111 (64.4)	
	Recessive	AA + GA	102 (87.7)	147 (82.6)	0.153
		GG	13 (11.3)	31 (17.4)	
	Additive	GG + AA	55 (47.8)	98 (55.1)	0.226
		GA	60 (52.2)	80 (44.9)	
		A	144 (62.6)	214 (60.3)	0.573
		G	86 (37.4)	141 (39.7)	
**rs2205796** (T > G)	Codominant	TT	59 (51.3)	117 (65.7)	**0.048**
		GT	49 (42.6)	54 (30.3)	
		GG	7 (6.1)	7 (3.9)	
	Dominant	TT	59 (51.3)	117 (65.7)	**0.014**
		GT + GG	56 (48.7)	61 (34.3)	
	Recessive	TT + GT	108 (93.9)	171 (96.1)	0.399
		GG	7 (6.1)	7 (3.9)	
	Additive	GG + TT	66 (57.4)	124 (69.7)	**0.032**
		GT	49 (42.6)	54 (30.3)	
		T	167 (72.6)	288 (80.9)	**0.019**
		G	63 (27.4)	68 (19.1)	
**rs909562** (A > G)	Codominant	AA	49 (42.6)	59 (33.1)	0.254
		GA	52 (45.2)	92 (51.7)	
		GG	14 (12.2)	27 (15.2)	
	Dominant	AA	49 (42.6)	59 (33.1)	0.101
		GA + GG	66 (57.4)	119 (66.9)	
	Recessive	GA + AA	101 (87.8)	151 (84.8)	0.471
		GG	14 (12.2)	27 (15.2)	
	Additive	GG + AA	63 (54.8)	86 (48.3)	0.280
		GA	52 (45.2)	92 (51.7)	
		A	150 (65.2)	210 (59.0)	0.130
		G	80 (34.8)	146 (41.0)	

Note: In the Han female participants, for rs2205796, there were significant differences in the genotypes and alleles distribution, dominant model and additive model between the case and control groups (*P* = 0.048, *P* = 0.019, *P* = 0.014, and *P* = 0.032, respectively)

**Table 4:** Distribution of SNPs of IDOL gene in Uygur male subjects

**Genotype**	**Model**		**Case (n, %)**	**Control (n, %)**	***P value***
**rs2072783** (A > G)	Codominant	AA	111 (49.0)	69 (49.6)	0.508
		GA	113 (45.7)	61 (43.9)	
		GG	23 (9.3)	9 (6.5)	
	Dominant	AA	111 (49.0)	69 (49.6)	0.374
		GA + GG	136 (55.1)	70 (50.4)	
	Recessive	AA + GA	224 (90.7)	130 (93.5)	0.332
		GG	23 (9.3)	9 (6.5)	
	Additive	GG + AA	134 (54.3)	78 (56.1)	0.724
		GA	113 (45.7)	61 (43.9)	
		A	335 (67.8)	199 (71.6)	0.276
		G	159 (32.2)	79 (28.4)	
**rs2205796** (T > G)	Codominant	TT	144 (58.3)	83 (59.7)	0.602
		GT	95 (38.5)	49 (35.3)	
		GG	8 (3.2)	7 (5.0)	
	Dominant	TT	144 (58.3)	83 (59.7)	0.787
		GT + GG	103 (41.7)	56 (40.3)	
	Recessive	TT + GT	239 (96.8)	132 (95.0)	0.380
		GG	8 (3.2)	7 (5.0)	
	Additive	GG + TT	152 (61.5)	90 (64.7)	0.531
		GT	95 (38.5)	49 (35.3)	
		T	383 (77.5)	222 (77.9)	0.906
		G	111 (22.5)	63 (22.1)	
**rs909562** (A > G)	Codominant	AA	123 (49.8)	74 (53.2)	0.755
		GA	102 (41.3)	52 (37.4)	
		GG	22 (8.9)	13 (9.4)	
	Dominant	AA	123 (49.8)	74 (53.2)	0.516
		GA + GG	124 (50.2)	65 (46.8)	
	Recessive	GA + AA	225 (91.1)	126 (90.6)	0.884
		GG	22 (8.9)	13 (9.4)	
	Additive	GG + AA	145 (58.7)	87 (62.6)	0.454
		GA	102 (41.3)	52 (37.4)	
		A	348 (70.4)	200 (71.9)	0.660
		G	146 (29.6)	78 (28.1)	

*Note*: In the Uygur male participants, there were no significant differences in the distributions of genotypes and alleles, dominant model, recessive model, and additive model for three SNPs between case and control groups (all *P* > 0.05)

**Table 5:** Distribution of SNPs of IDOL gene in Uygur female subjects

**Genotype**	**Model**		**Case (n, %)**	**Control (n, %)**	***P value***
**rs2072783** (A > G)	Codominant	AA	31 (54.4)	84 (46.9)	0.405
		GA	22 (38.6)	72 (40.2)	
		GG	4 (7.0)	23 (12.8)	
	Dominant	AA	31 (54.4)	84 (46.9)	0.327
		GA + GG	26 (45.6)	95 (53.1)	
	Recessive	AA + GA	53 (93.0)	156 (87.2)	0.332
		GG	4 (7.0)	23 (12.8)	
	Additive	GG + AA	35 (61.4)	107 (59.8)	0.827
		GA	22 (38.6)	72 (40.2)	
		A	84 (73.7)	240 (67.0)	0.183
		G	30 (26.3)	118 (33.0)	
**rs2205796** (T > G)	Codominant	TT	36 (63.2)	116 (64.8)	0.975
		GT	19 (33.3)	57 (31.8)	
		GG	2 (3.5)	6 (3.4)	
	Dominant	TT	36 (63.2)	116 (64.8)	0.821
		GT + GG	21 (36.8)	63 (35.2)	
	Recessive	TT + GT	55 (96.5)	173 (96.6)	0.955
		GG	2 (3.5)	6 (3.4)	
	Additive	GG + TT	38 (66.7)	122 (68.2)	0.834
		GT	19 (33.3)	57 (31.8)	
		T	91 (79.8)	289 (80.7)	0.832
		G	23 (20.2)	69 (19.3)	
**rs909562** (A > G)	Codominant	AA	33 (57.9)	86 (48.0)	0.354
		GA	21 (36.8)	76 (45.2)	
		GG	3 (5.3)	17 (9.5)	
	Dominant	AA	33 (57.9)	86 (48.0)	0.195
		GA + GG	24 (42.1)	93 (52.0)	
	Recessive	GA + AA	54 (94.7)	162 (90.5)	0.317
		GG	3 (5.3)	17 (9.5)	
	Additive	GG + AA	36 (63.2)	103 (57.5)	0.453
		GA	21 (36.8)	76 (45.2)	
		A	87 (76.3)	248 (69.3)	0.149
		G	27 (23.7)	110 (30.7)	

*Note*: In the Uygur female participants, there were no significant differences in the distributions of genotypes and alleles, dominant model, recessive model, and additive model for three SNPs between case and control groups (all *P* > 0.05)

Table 6 and 7 showed the multivariable logistic regression analyses of the major risk factors for CAD in Han and Uygur ethnic groups by different genders. According to the results of the multivariate adjustments for the confounders such as age, smoking, drinking, hypertension, diabetes, TG, TC, HDL-C, LDL-C, and FPG, in the Han female subjects, the rs2205796 SNP is an independent risk factor for CAD [TT vs. GG/GT: odds ratio = 2.304, 95% confidence interval = 1.211–4.83, *P* = 0.011]. Whereas after adjustment for other confounders, rs2072783 and rs909562 SNPs are not the independent risk factors for CAD (*P* = 0.695 and *P* = 0.555). The three SNPs do not represent as the independent risk factors for CAD in the Han male participants and the Uygur population (all *P* > 0.05).

**Table 6:** Results of logistic analysis in male subjects

**Groups**	rs2072783	rs2205796	rs909562	Age	Smoking	Drinking	Hypertension	Diabetes	TG	TC	HDL-C	LDL-C	FPG
Han													
OR	0.938	1.430	0.923	1.028	0.752	1.319	1.588	2.402	1.120	1.263	0.135	1.490	0.910
95% CI	0.578–1.520	0.889–2.299	0.569–1.495	1.008–1.049	0.456–1.242	0.818–2.128	0.996–2.533	0.969–5.945	0.951–1.318	0.976–1.571	0.068–0.270	1.141–1.946	0.783–1.057
*P* value	0.794	0.140	0.743	0.006	0.266	0.256	0.052	0.058	0.174	< 0.001	< 0.001	0.003	0.215
Uygur													
OR	1.224	1.132	0.901	1.402	0.833	4.163	1.425	0.349	1.254	0.834	0.414	0.933	1.386
95% CI	0.690–2.171	0.688–1.860	0.502–1.617	1.015–1.071	0.496–1.397	2.357–7.355	0.867–2.341	0.105–1.159	0.974–1.616	0.659–1.054	0.243–0.706	0.755–1.154	1.090–1.763
* P* value	0.489	0.624	0.726	0.002	0.488	< 0.001	0.162	0.086	0.080	0.129	0.001	0.524	0.008

*Note*: In the Han and Uygur male participants, the three SNPs did not represent as the risk factor of CAD (all *P* > 0.05)

*TC* total cholesterol, *TG* triglyceride, *HDL-C* high-density lipoprotein cholesterol, *LDL-C* low-density lipoprotein cholesterol, *FPG* fasting plasma glucose

**Table 7:** Results of logistic analysis in female subjects

**Groups**	rs2072783	rs2205796	rs909562	Age	Smoking	Drinking	Hypertension	Diabetes	TG	TC	HDL-C	LDL-C	FPG
Han													
OR	1.150	2.304	0.811	1.078	0.196	2.468	1.189	3.102	1.458	0.710	0.830	0.950	1.175
95% CI	0.572–2.314	1.211–4.834	0.404–1.682	1.046–1.111	0.037–1.043	1.664–3.662	0.626–2.258	0.050–14.799	1.000–2.125	0.496–1.016	0.466–1.481	0.719–1.255	0.868–1.593
* P* value	0.695	0.011	0.555	< 0.001	0.056	< 0.001	0.597	0.156	0.050	0.061	0.529	0.716	0.297
Uygur													
OR	1.038	0.532	0.981	1.128	1.512	6.732	1.425	2.177	1.141	0.696	0.024	2.179	1.252
95% CI	0.348–3.100	0.182–1.552	0.321–2.998	1.061–1.200	0.041–56.00	3.891–11.646	0.564–3.600	0.324–14.624	0.788–1.653	0.308–0.799	0.004–0.148	1.358–3.497	0.949–1.651
* P* value	0.946	0.894	0.973	< 0.001	0.823	< 0.001	0.177	0.423	0.485	0.073	< 0.001	0.001	0.111

*Note*: In the Han female subjects, the rs2205796 SNP represents as an independent risk factor for CAD (*P* = 0.011). In the Uygur female participants, the three SNPs did not represent as the risk factor of CAD (all *P* > 0.05)

*TC* total cholesterol, *TG* triglyceride, *HDL-C* high-density lipoprotein cholesterol, *LDL-C* low-density lipoprotein cholesterol, *FPG* fasting plasma glucose

## Discussion

In the present study, we investigated the relationships between three SNPs in the human IDOL gene and risk factors of CAD in Han and Uygur populations. This was the first attempt to study some common variants in the IDOL gene and their correlations with CAD in these populations. Our results indicated that rs2205796 was strongly correlated with CAD susceptibility in the Han female population.

The human IDOL gene is located in 6p23–p22.3 [19], and it encodes a 445 amino acid protein identified as an E3-ubiquitin ligase. The E3-ubiquitin ligase contains the N-terminal frame of ezrin/radixin/moesin homology (FERM) domain and the C-terminal catalytic of really interesting new gene (RING) domain, and they are separated by a short linker region [20]. IDOL functions can be regarded as the regulator for cellular cholesterol uptake with the help of the LDL receptor (LDLR) pathway [6], and is the direct target regulated by the nuclear receptor liver X receptor (LXR). Meanwhile, IDOL expression is coordinately regulated by the ATP-binding cassette transporter in multiple cell types [21]. In response to cellular cholesterol loading, activation of LXR leads to the rapid induction of IDOL expression. IDOL conducts the degradation of LDLR by means of stimulating ubiquitination of the LDLR on its cytoplasmic tail. IDOL expression in mouse liver can markedly decrease the levels of LDLR protein and then increase the levels of LDL-C [22, 23]. Therefore, the LXR-IDOL-LDLR pathway can be regarded as a complementary pathway for sterol regulatory element-binding proteins to realize the feedback inhibition of cholesterol uptake.

The relationship between plasma lipid profiles and the IDOL gene polymorphisms was poor, and previous studies showed inconsistent results. Adi et al. [24] reported that the rs149696224 SNP in the IDOL gene was related to the high LDL-C levels in the Chinese Uygur population. Their study showed the G51S mutation stabilizes IDOL protein through inhibiting its dimerization and self-ubiquitination and, consequently, leading to increased LDLR degradation. A study performed by Yan et al. [25] analyzed the relevance of plasma lipid levels and the rs3757354 SNP of the human IDOL gene in different ethnic groups. They found that the serum lipid levels and IDOL rs3757354 SNP in Han group differs from other minority ethnic groups. Ashish et al. [26] reported that the N342S variant of the IDOL gene had no impact on plasma lipid profiles, and no relevance with CVD and atherosclerosis progression in the general Italian population.

In our present study, we carried out two independent case–control studies to investigate the relevance of CAD and IDOL gene polymorphisms, and found that the rs2205796 was related to CAD in the Han female population. Further, the Logistic analysis results showed that the relevance of rs2205796 SNP and CAD remains significant after adjustment for several confounders. However, the results we mentioned were only found in Chinese the Han female population, but not taken from the Han male and the Uygur population. There may be some reasons resulting in above differences. Xinjiang is a province with vast territory and diverse ethnic customs. Besides the different genetic backgrounds in the two ethnic groups, the influence of ecological environment, the different living habits and lifestyles may play a role in it. The Uygur population consume more pasta, meat and milk products than the Han population do, and the Han people eat more rice, vegetables and fruits than other minorities do. Our study indicated that the Chinese Han female population with rs2205796 SNP of IDOL gene may have increased susceptibility to CAD. Our results may help scientific researchers and doctors working on CAD improve the screening quality and early diagnosis of coronary heart disease in this population. The results may be helpful for the early prevention of public health problems such as cardiovascular disease, in this population in Xinjiang.

There are several limitations in our study. First of all, our conclusion drew only by the present observational study, and it lacked functional validation. Secondly, the study population is only from one hospital, which may produce selective bias. Finally, the sample size of our study was still small and based on only one center. Large sample and multi center researches are still needed to confirm our conclusions.

## Conclusions

In summary, the rs2205796 polymorphism of the IDOL gene is associated with CAD in the Chinese Han female population. Subjects with GG/GT genotype or G allele of rs2205796 were related to an increased risk of CAD.

## Data Availability

The data will not be shared, since part of the data is being reused by another study.

## Abbreviations

CAD: Coronary artery disease
HDL: High-density lipoprotein
LDL: Low-density lipoprotein
TC: Total cholesterol
TG: Triglyceride
