# Correction to: Genetic polymorphism of IDOL gene was associated with the susceptibility of coronary artery disease in Han population in Xinjiang, China

**DOI:** 10.1186/s41065-021-00185-x

**Published:** 2021-06-05

**Authors:** Dilare Adi, Jialin Abuzhalihan, Jing Tao, Yun Wu, Ying-Hong Wang, Fen Liu, Yi-Ning Yang, Xiang Ma, Xiao-Mei Li, Xiang Xie, Zhen-Yan Fu, Yi-Tong Ma

**Affiliations:** 1 Department of Cardiology, First Affiliated Hospital of Xinjiang Medical University, Urumqi, 830054 PR China; 2 Xinjiang Key Laboratory of Cardiovascular Disease Research, Urumqi, 830054 PR China; 3 People’s Hospital of Xinjiang Uygur Autonomous Region, Urumqi, 830001 PR China; 4 Department of General Practice, First Affiliated Hospital of Xinjiang Medical University, Urumqi, 830054 PR China; 5 Health Checkup Department of The First Affiliated Hospital of Xinjiang Medical University, Urumqi, 830054 PR China; 6 State Key Laboratory of Pathogenesis, Prevention and Treatment of High Incidence Diseases in Central Asia, Clinical Medical Research Institute, the First Affiliated Hospital of Xinjiang Medical University, Urumqi, 830054 PR China

**Correction to: Hereditas 158, 12 (2021)**

**https://doi.org/10.1186/s41065-021-00178-w**

Following the publication of the original article [1], it was noted that the following equal contribution note was missing.

Dilare Adi and Jialin Abuzhalihan contributed equally to this work.

The original article has been updated.
